# Supplementary figures and images for: Analysis of gut microbiota and the effect of lauric acid against necrotic enteritis in Clostridium perfringens and Eimeria side-by-side challenge model
Source: PLoS One. 2019 May 31;14(5):e0205784. doi: 10.1371/journal.pone.0205784 (PMC6544216; doi:10.1371/journal.pone.0205784)

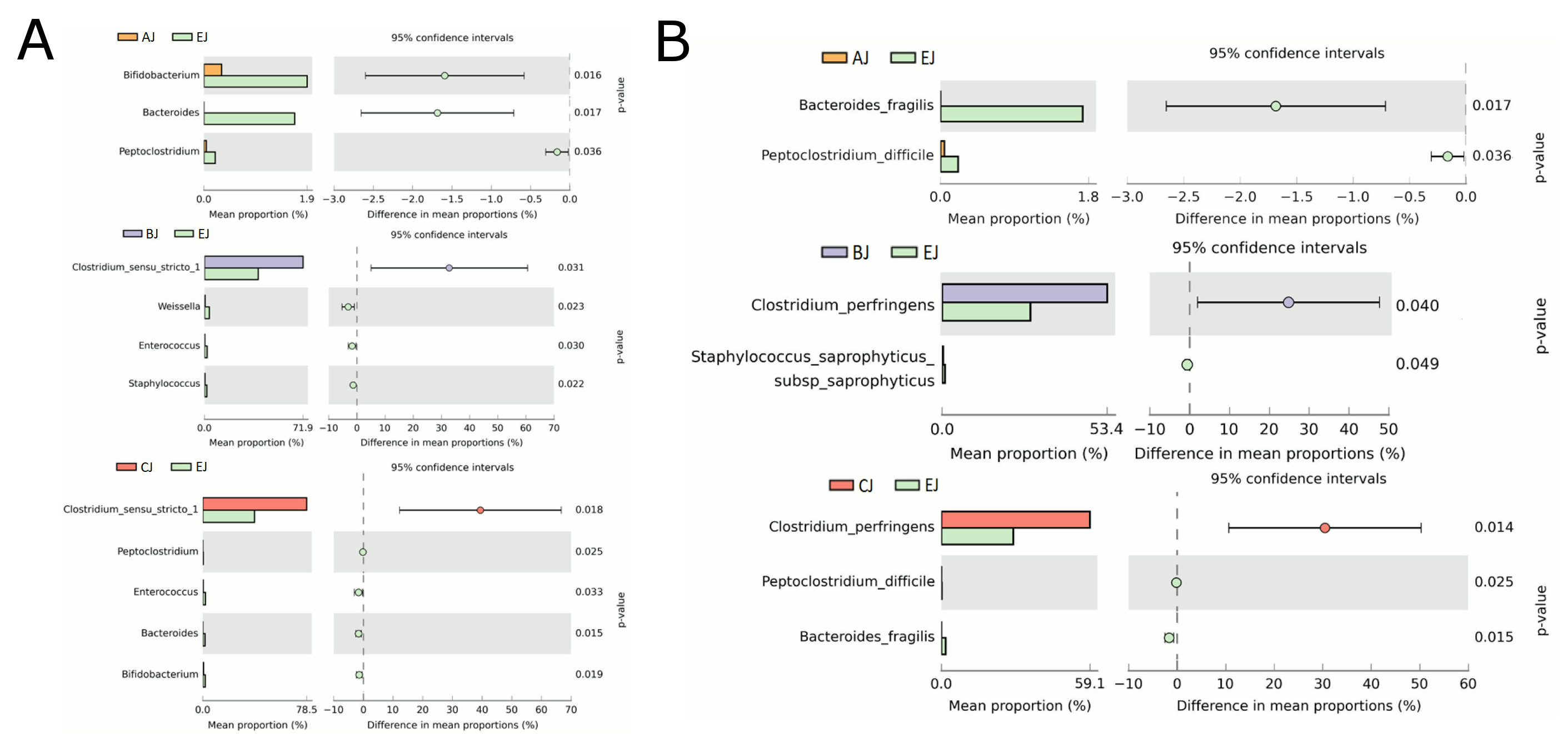

Supplement: S5 Fig — Significant difference at the genus (A) and species (B) level by STAMP with Welch's t-test. The different genera or species were presented only to those presenting a minimum variation at a significant level (p < 0.05). Mutual comparisons were performed between AJ and EJ, BJ and EJ, and CJ and EJ groups. The abundance of Clostridium sensu stricto 1 and C. perfringens in CP1+Eimeria (BJ) and lauric acid supplementation (CJ) had highly significant abundance compared to the control (EJ) in jejunal microbiota. (TIF) [file pone.0205784.s005.tif]
